# A Novel Approach to Charcoal Fine Waste: Sustainable Use as Filling of Polymeric Matrices

**DOI:** 10.3390/polym14245525

**Published:** 2022-12-16

**Authors:** Fabíola Martins Delatorre, Gabriela Fontes Mayrinck Cupertino, Michel Picanço Oliveira, Felipe da Silva Gomes, Luciene Paula Roberto Profeti, Demetrius Profeti, Mário Guimarães Júnior, Márcia Giardinieri de Azevedo, Daniel Saloni, Ananias Francisco Dias Júnior

**Affiliations:** 1 Agricultural Sciences and Engineering Center, Federal University Espírito Santo, Jerônimo Monteiro 29550-000, Espiríto Santo, Brazil; 2 Department of Eletromechanical, Federal Center of Technological Education of Minas Gerais, Araxá 38180-510, Minas Gerais, Brazil; 3 Advanced Materials Laboratory, North Fluminense University State Darcy Ribeiro, Campos dos Goytacazes 28013-602, Rio de Janeiro, Brazil; 4 Department of Forest Biomaterials, College of Natural Resources, North Carolina State University, Raleigh, NC 27695, USA

**Keywords:** carbon materials, waste reuse, forest-based biomaterials, XPS

## Abstract

Most composites produced come from fossil fuel sources. Renewable strategies are needed for the production of composites. Charcoal fines are considered waste and an alternative for the production of biocomposites. The charcoal fines resulting from the pyrolysis of any biomass are an efficient alternative for the production of green composites. Studies to understand how the pyrolysis parameters influence the properties of this material for the production of biocomposites are necessary. Charcoal has a high carbon content and surface area, depending on final production temperatures. This study aims to evaluate charcoal fines as potential reinforcing agents in biocomposites. This study investigated for the first time charcoal fines from three pyrolysis temperatures (400, 600, and 800 °C) to identify the most suitable charcoal for use as a raw material in the production of carbon biocomposites with 30% by weight incorporated into a polyester matrix composite. Apparent density, porosity, morphology, and immediate chemical composition and Fourier transform infrared spectroscopy (FTIR) and X-ray photoelectron spectroscopy (XPS) of charcoal fines were evaluated. The charcoal fines produced at 800 °C showed interesting potential as polymeric matrix fillers due to their higher porosity (81.08%), fixed carbon content (96.77%), and hydrophobicity. The biocomposites were analyzed for flexural and tensile strength and scanning electron microscopy. The results revealed an improvement in resistance at elevated temperatures, especially at 800 °C, with higher breaking strength (84.11 MPa), modulus of elasticity (4064.70 MPa), and traction (23.53 MPa). Scanning electron microscopy revealed an improvement in morphology, with a decrease in roughness at 800 °C, which caused greater adhesion to the polyester matrix. These results revealed a promising new biocomposite compared to other natural lignocellulosic polymeric composites (NLFs) in engineering applications.

## 1. Introduction

Studies have sought to develop new materials to meet current technological demands and industrial sectors with low environmental impact. Thus, science has proposed utilizing industrial waste as raw materials for other processes, promoting the use of resources and ensuring sustainability. Charcoal is a highly friable material, produced in copious quantities by the thermochemical conversion of plant biomass, resulting in enormous amounts of fines (>25% of the production) [1]. These residues usually lack adequate disposal, a problem for manufacturers in developing countries such as Brazil, especially for manufacturers. We propose using these residues to create new composite materials, such as reinforcing agents in polymeric, thermoplastic, and/or rigid biocomposites used in various industrial applications. We base this hypothesis on its high carbon constitution, porosity, surface area [2,3], and the fact that we can control the pyrolysis process to obtain these specific properties. We aimed to evaluate the most appropriate parameters to develop new polymeric biocomposites in a matrix with charcoal fines.

Pyrolysis occurs in controlled amounts of oxygen at temperatures above 300 °C, and factors such as permanence in reaction zones, final temperature, pressure, and heating rate influence it [1,4]. Thus, we need to investigate the relevant parameters originating charcoal, especially the absolute temperature, since it affects all crucial characteristics for producing carbon biocomposite [5,6]. 

Polymer production consists mainly of fossil fuels, severely impacting the environment due to these materials’ chemical, physical, and biological inertia [2]. Research aimed at producing new sustainable materials to circumvent this impact has intensified [7]. However, studies on synthesizing polymeric materials with charcoal fines additives are still emergent. This material is an alternative to polymeric products of purely fossil origin, which could add value to this abundant and renewable biomass.

Researchers proposed the first studies analyzing the potential of charcoal to produce polymeric biocomposites [2,3,8]. They analyzed charcoal-filled composites and their thermal, mechanical, and electrical properties to characterize their composition and behavior. These studies attest to the beneficial effects of adding charcoal to polymeric matrices to reinforce their properties. The abundant production of charcoal as an energy source and other applications may suit its potential use as an additive. Studies are needed to evaluate the use of charcoal fines as additives, as they are residues that need to be reinserted into the production chain to minimize their life cycle and add value to production. Charcoal depends on pyrolysis temperature, which alters its characteristics for use as fillers of polymeric matrices. Managing pyrolysis temperatures in order to use fines in biocomposite production requires an urgent resolution for developing new carbon products.

Charcoal fines resulting from the pyrolysis of any biomass are an efficient alternative for producing sustainable composites since their carbon, porous, and hydrophobic structure allows more significant interaction with polymer matrices [3]. This study evaluates the physical and chemical properties of charcoal fines produced at different pyrolysis temperatures and how these characteristics achieved the mechanical properties of the carbon biocomposites produced.

## 2. Materials and Methods

### 2.1. Biomass Characterization and Charcoal Production

**Figure A1:**
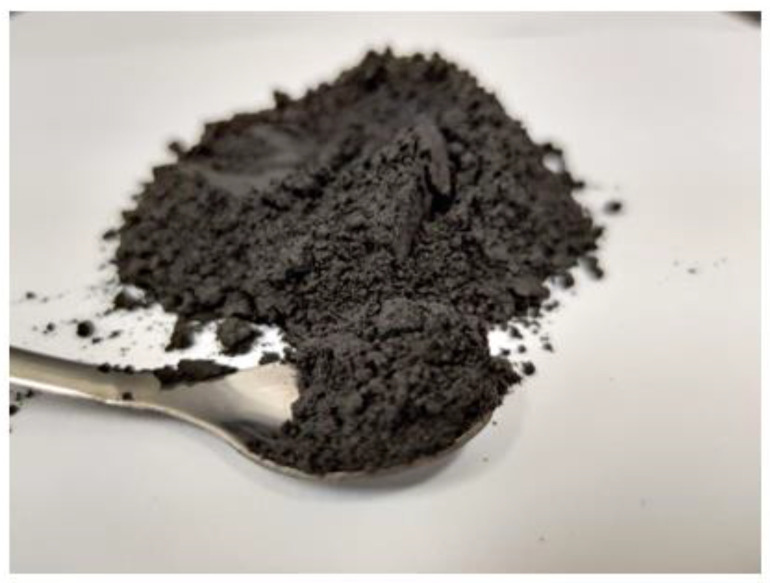
Demonstration of the charcoal fines produced.

Chips from 10-year old *Eucalyptus saligna* trees were used as raw materials for charcoal fines (Appendix A, Figure A1). Basic density [9], chemical composition of extractives, lignin [10], hemicelluloses [11], ash content [12], and porosity [13] were measured.

Then, the material was pyrolyzed. Wood dried in a 103 ± 2 °C oven was charred in a reactor inside a muffle whose inert atmosphere was obtained by the continuous insertion of nitrogen gas (3 mL·min^−1^). Chips were pyrolyzed at 400, 600, and 800 °C, at a heating rate of 10 °C·min^−1^, beginning at 30 °C until they reached their final temperature, in which they remained for 120 min of conditioning. After each process, the charcoal mass was measured, and the pyroligneous liquid obtained by gas condensation was evaluated for its mass and volume. Thus, the gravimetric charcoal, pyroligneous liquid, and noncondensable gas yields were estimated in relation to the initial wood chip mass via Equations (1)–(3).

(1) $$\mathrm{CY}\;=\left(\frac{\mathrm{Cm}}{\mathrm{Dm}}\right)\times 100$$

(2) $$\mathrm{PLY}\;=\left(\frac{\mathrm{PLm}}{\mathrm{Dm}}\right)\times 100$$

(3) NCGY =100−(CY + PLY)

where: CY = charcoal yield (%); Cm = charcoal mass (g); Dm = dry wood mass (g); PLY = pyroligneous liquid yield (%); PLm = pyroligneous liquid mass (g); NCGY = noncondensable gas yield (%).

#### 2.1.1. Charcoal Fine Characterization

Apparent density was estimated by dividing the sample mass by its volume (Apd = mass/volume, in g·cm^−3^), porosity [13] and the determination of immediate chemical analysis (volatile material, ash and fixed carbon) [14].

#### 2.1.2. Visual Analysis of Charcoal Microstructures

This analysis was performed in two steps. The first step involved the aid of the Olympus LEXT Confocal Microscope—3D Measuring L. Microscope 4000 to assess degradation or maintenance of solid charcoal in chips prior to maceration. Images were obtained via an objective 50× magnification lens (0.95 numerical aperture; with a 0.26 mm × 0.26 mm field of view, and about 0.25 μm sampling distance) and an objective 100× magnification lens (0.95 numerical aperture, with a 0.13 mm × 0.13 mm working field of view, and a 0.13 μm sampling distance). 405 nm light beam wavelength. The OLS4000 2.1 software was used to treat the images. Then, charcoal fines were analyzed (250 mesh—0.056 mm). The fixation of the material on a metal support with carbon tape and metallized with gold in the Balzers Union SCD 030 system ensured the accurate scanning of secondary electrons during microscopy using a scanning electron microscope (Jeol, model JSM-IT200, Peabody, MA, USA) operating at 10 kV. SEM images were obtained using the proprietary JEOL software.

#### 2.1.3. Fourier-Transform Infrared Spectroscopy (FTIR)

To characterize the aromatic structures (functional groups and chemical bonds) of charcoal fines, a spectroscopy analysis was performed in the Fourier-transform infrared region (FTIR).

#### 2.1.4. Charcoal Fine X-ray Photoelectron Spectroscopy

An X-ray excited photoelectron (XPS) spectrometer, model K-Alpha, from Thermo Scientific at the National Nanotechnology Laboratory (LNNano) at the National Center for Research in Energy and Materials (CNPEM, Brazil) was used for energy analysis of surface of charcoal fines. A monochromatic Al Kα (1486.6 eV) X-ray source of 300 W was used at a 30° take-off angle to the sample surface. Measurements were taken in a 9 × 9 mm^2^ area under a 5 × 10^−10^ mbar high vacuum at room temperature. Spectra were obtained in bond energies between 0 and 1150 eV via three scans with 160 eV passing energy and 1 eV resolution. 

### 2.2. Production and Characterization of Biocomposites

The charcoal fines were ground in an MA-500 ball mill for three hours and then homogenized in a 250 mesh sieve (0.056 mm) (Appendix A, Figure A1) according to the conditions proposed in this study, thus simulating the fines obtained in industrial operations. The charcoal fines were dried in an oven with air circulation at 103 ± 2 °C for 24 h. The polymeric matrix used was polyester resin (UC 2120 AC PLUS), with molecular weight Mn = 9 × 10^3^ g·mol^−1^, as well as the butanox catalyst, supplied by Redelease (São Paulo, Brazil). The biocomposites were developed using polyester resin to verify the feasibility of using charcoal fines as filler in the polymeric matrix. The biocomposites were produced in the proportion of 30% of charcoal fines at different final temperatures of charcoal pyrolysis (400, 600, and 800 °C). Catalyst was added to 2% resin by weight, as suggested by the manufacturer. Subsequently, the mixture of the fines and the polymeric matrix was carried out in a mechanical stirrer for three minutes for better homogenization of the samples. The mixture was poured into the silicone mold and placed in a compressed air reactor from the company MM (Lavras, Brazil). After 24 h of curing under the pressure of 90 bar, complete polymerization occurred. 

#### 2.2.1. Mechanical Tests 

**Figure A2:**
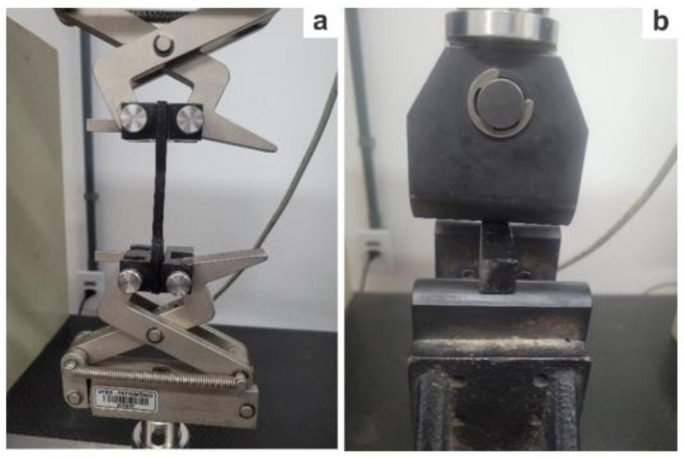
(**a**) Flexural strength test (**b**) and tensile strength test of biocomposites.

Mechanical tests were performed to verify the properties of this material. The bending and tensile tests were performed using a universal mechanical testing machine model EMIC, the bending strength (Appendix A, Figure A2a) was defined following the parameters of the ASTM D-7264 standard [15] and the tensile tests (Appendix A, Figure A2b) followed the prescriptions of the ASTM D-3039 standard [16]. Seven samples were tested for each trial to overcome experimental and instrumental errors.

#### 2.2.2. Fractographic Analyses 

Then, the charcoal fines were analyzed (250 mesh—0.056 mm). The fixation of the material on a metal support with carbon tape and metallized with gold in the Balzers Union SCD 030 system ensured the accurate scanning of secondary electrons during microscopy using a scanning electron microscope (Jeol, model JSM-IT200, Peabody, MA, USA), equipped with an energy disperser. The images were obtained using the proprietary Jeol software.

### 2.3. Data Analysis

Data were provided for normality (Shapiro–Wilk) and homoscedasticity (Bartlett) tests. Analysis of variance was performed following a completely randomized design, with three response variables related to the pyrolysis temperature (400, 600 and 800 °C), with seven replications using composites with 30% charcoal fines. After detecting significant differences, the regression model that best predicted the behavior of the data was adjusted. All analyses were performed at 95% probability. Standard error measures were provided to better understand the confidence interval obtained for each variable studied. The R core Team software was used for all statistical analysis.

## 3. Results and Discussion

**Figure A3:**
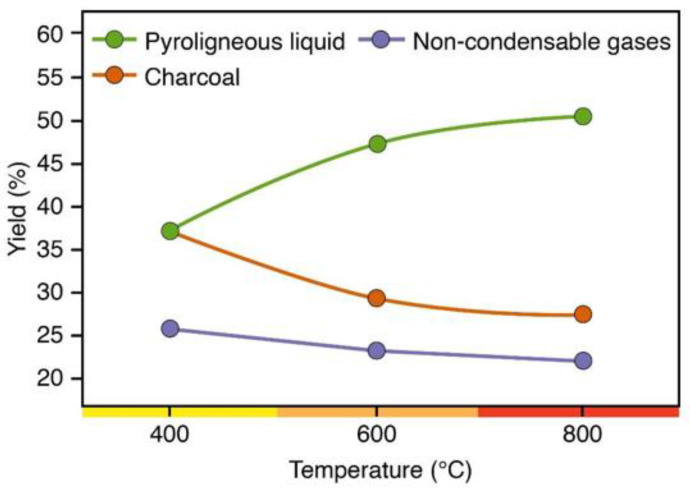
Yield of charcoal fines.

*E. saligna* charcoal fines showed the following average values: basic density = 0.57 g·cm^−3^; extractives = 5.79%; lignin = 25.8%; ash = 0.15%, and porosity = 68.22%. These properties relate directly to pyrolysis products. Basic density and lignin content are relevant for charcoal production, increasing charcoal volumetric and gravimetric yields and favoring greater biocomposite manufacture [17,18]. Yield also depends on pyrolysis variables, such as reaction atmosphere, heating rate, and, especially, final temperature (Appendix A, Figure A3). 

The results show that 400 °C pyrolysis obtained the highest yield, 37.15%, whereas 800 °C, the lowest, 27.42%, results inversely proportional to pyroligneous liquid yield. Figure A3 shows that non-condensable gas yields failed vary significantly vary with increasing temperatures (Appendix A): 25.7, 23.27, and 22.04% in 400, 600 and 800 °C, respectively. To optimize production, greater charcoal yields favor biocomposite filling due to larger volumes of the material of interest. However, its chemical and physical properties will effectively contribute to the synergy of the materials involved in producing carbon biocomposites [19]. 

**Figure 1:**
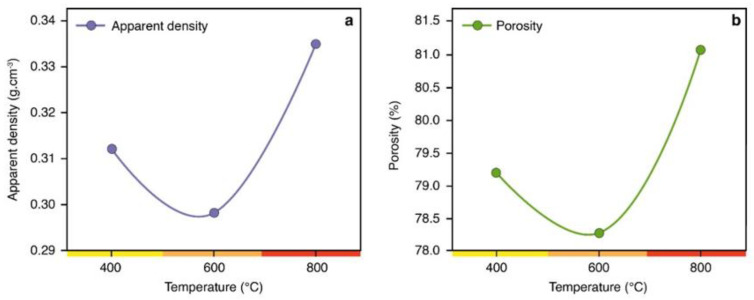
(**a**) Apparent density and (**b**) porosity of charcoal fines produced at different pyrolysis temperatures.

A higher final pyrolysis temperature (600 to 800 °C) increased in 7% the apparent density of charcoal (Figure 1A). This behavior is due to intense hydrogen output, an element capable of strong molecular bonds absorbing significant energy in pyrolysis above 500 °C [20]. This relates to the rearrangement of the chemical structure of biomass under heat, producing a graphitic structure that benefits the mechanical resistance of the material [21,22]. By proposing charcoal as a reinforcing agent in polymeric matrices, we expect increased mechanical strength and developed density in the new material. We hope biocomposites filled with high-temperature charcoal will show higher strength, facilitating their use in structural applications. 

Porosity is another relevant parameter for producing biocomposites. Charcoal is highly porous (up to 85% of its volume) and its pore sizes range from sub-nanometers to tens of micrometers, depending on raw materials and pyrolysis temperatures [23,24]. Figure 1b shows that higher temperatures result in a more porous charcoal—800 °C pyrolysis favored their formation. Studies state that the progressive removal of volatile materials from charcoal pores, increased connection of existing pores, and condensation of the remaining skeletal structure cause this behavior [23,24,25,26]. 

**Figure 2:**
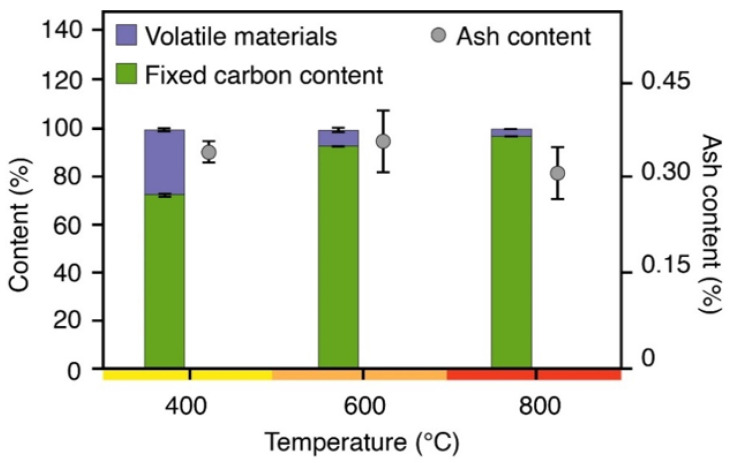
Immediate chemical composition of charcoal fines. Source: Where: FC = fixed carbon content; VM = volatile material content; AS = ash content.

Their immediate chemical composition (Figure 2) shows that higher pyrolysis temperatures increase the percentage of fixed carbon and, proportionally, the reduction of volatile materials. Evaluating the immediate chemical composition of charcoal fines as a function of pyrolysis temperature is important for predicting biocomposite performance [27]. Studies discuss that materials with a higher carbon content provide greater mechanical strength, which can positively increase the resistance of the biocomposites produced [28,29]. We observe a decrease in the ash content of charcoal produced at 800 °C, which may enable a greater interaction between polymeric matrices and charcoal fines due to its high fixed carbon and lower ash contents. We expect these characteristics to provide biocomposites with greater interaction and mechanical resistance. 

**Figure 3:**
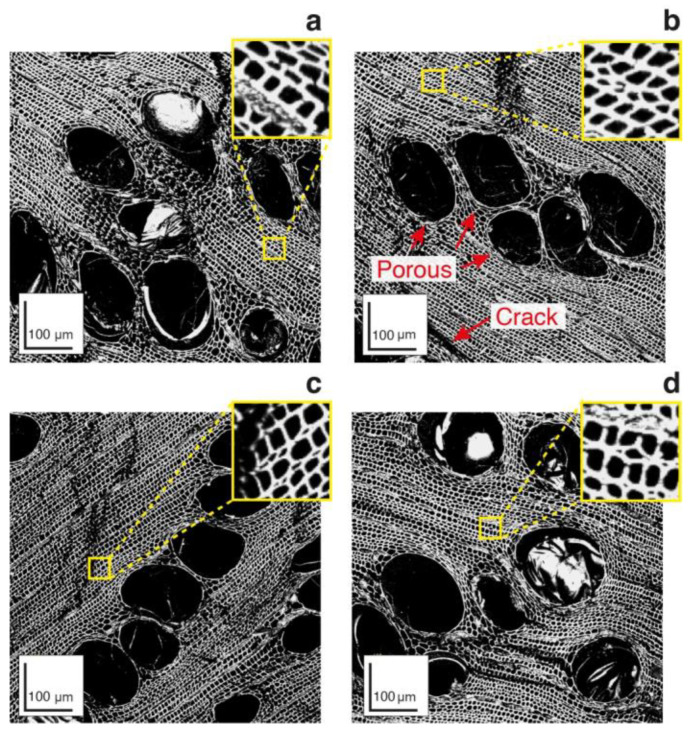
Confocal microscopy of the anatomical transverse structure of charcoal produced at 400 °C (**a**,**b**), 600 °C (**c**), and 800 °C (**d**).

Confocal microscopy images show increased charcoal porosity at higher temperatures (Figure 3). Through the SEM images and the percentage of pores in Figure 1b, there was an increase of 2.37 and 3.58%, generally of a nonpolar nature [28,29] of pores compared to charcoal at 800 °C at temperatures 400 and 600 °C, respectively. These characteristics are relevant for using the material as reinforcing agents in biocomposites since they can promote greater adhesion to polymeric matrices, better wetting, and greater mechanical resistance by high impregnation and better spreadability. The literature indicates that a more porous structure allows polymeric resins to fuse and entangle with charcoal, creating a strong interfacial support between fillings and matrices, resulting in improved properties, especially mechanical resistance [28,30,31]. However, polymer penetration into charcoal pores will depend on the viscosity of the polymer resin and pore size [30]. 

**Figure 4:**
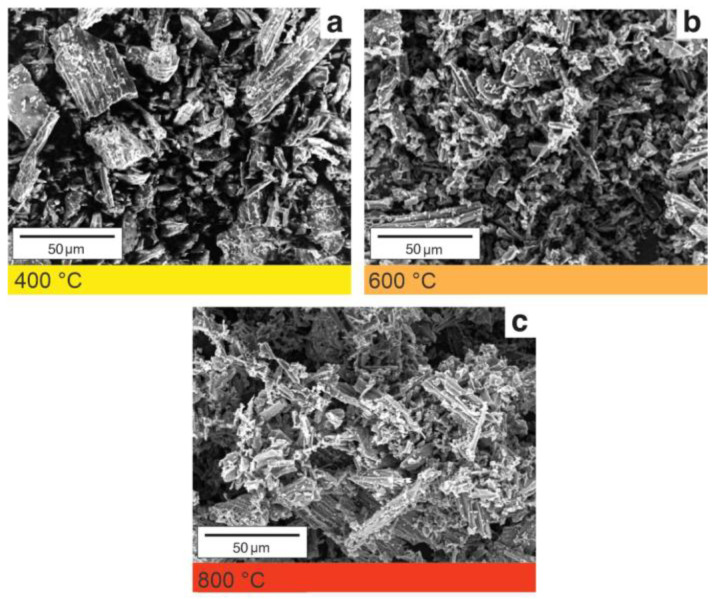
Micrographs obtained by scanning electron microscopy of charcoal fine samples synthesized at 400 (**a**), 600 (**b**), and 800 °C (**c**) in ×300 magnification.

We highlight that such aspects depend on particle dimensions of charcoal fines used as filler. Figure 4 shows the SEM images of charcoal fines produced at different final temperatures. Charcoal fines have a rough surface, due to pores collapsing and ash filling the porous system. Different temperatures did not produce significant morphological differences, which would require higher heating rates. Despite homogenization, we observe charcoal fines of varied sizes. Pyrolysis conditions influence surface morphology and the physical properties of charcoal fines. 

**Figure 5:**
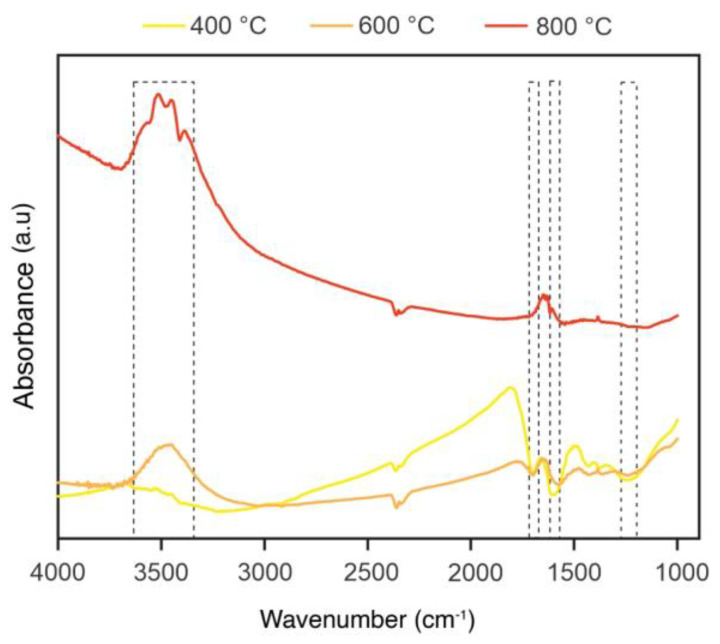
FTIR spectra of charcoal fines produced at different pyrolysis temperatures.

Figure 5 shows the FTIR spectra of charcoal fines produced at three pyrolysis temperatures and changes to their chemical structure. The stretching vibration of the asymmetric OH group—referring to phenol, alcohol, carboxylic acid groups, and water—formed a 3663 cm^−1^ broad peak. The 1671 and 1728 cm^−1^ peaks refer to the vibration and elongation of the CO double bond, whereas the 1500 cm^−1^ peaks, to the vibration in the double bond between aromatic carbons with olefins and aromatic structures. On the other hand, 1180 to 1297 cm^−1^ peaks relate to elongating vibrations of the C-O connection. Figure 5 shows that higher temperatures affected functional groups, changing charcoal structures. The formation of hydroxyl groups at 800 °C favors the elevated polarity of the material, profoundly influencing charcoal and polymer matrix interaction in composites [32], essential for good mechanical properties [32,33,34]. Moreover, the free hydroxyl groups in charcoal produced at 800 °C allow greater interaction between materials, contributing to the generation of highly compatible composites [2,3].

**Figure 6:**
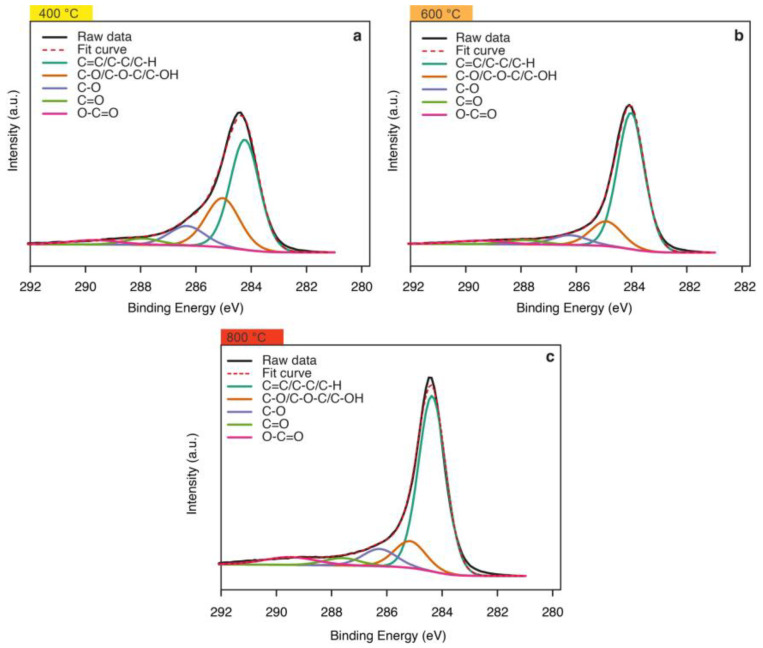
X-ray photoelectron spectroscopy (XPS) of charcoals at temperatures of 400 (**a**), 600 (**b**), and 800 °C (**c**).

We performed an XPS analysis to understand how the chemical changes to charcoal can contribute to resin interaction. Figure 6 shows the deconvolution of the XPS spectra of charcoal fine samples. Note the spectral region comprising the binding energies characteristic of C1s in the materials synthesized at 400 (Figure 6a), 600 (Figure 6b), and 800 °C (Figure 6c). Analysis of C1s spectra showed five peaks related to the different carbon species in charcoal fines. The most intense peak (EB = ~284.0 eV) corresponds to functional states of species with C=C or C−sp2 bonds in aromatic/allophatic carbons [34]. Due to the nature of the atoms surrounding these species, the binding energy in this peak may undergo small variations due to changes in pyrolysis temperature, since heating affected the chemical composition of the surface [35]. 

Higher pyrolysis temperatures changed the structure and chemical nature of charcoal; attested by the increased intensity of peaks of aromatic/allophatic groups in XPS spectra. As expected, heating develops more compact aromatic carbon structures and more intense spectral peaks. On the other hand, higher temperatures decrease the proportion of functional groups formed by carbon atoms and heteroatoms, especially oxygen, in the composition of charcoal fines [36]. Table 1 shows this aspect by comparing the mean relative percentages related to the contribution of each carbon species to the total composition of the material. 

Analyzing XPS spectra shows the majority presence of aromatic or aliphatic carbon species, and C−/C−O−C/C−OH groups (ether, phenol, etc.), C−O (ketone), C=O (carbonyl) and O−C=O (carboxylic acid, ester), located at 284.70 eV, 286.15 eV, 287.75 eV, and 288.80 eV binding energies, respectively [35,36]. These functional carbon and oxygen groups comprise a smaller proportion of the composition, tending to decrease as temperatures rose from 400 to 600 °C. However, they remained stable from 600 °C upward since we observed no significant variations in percentage contributions as temperatures neared 800 °C.

Note in the XPS spectra (Figure 6) that the energy of the C−C/C−H group dominates the charcoal composition, intensifying as temperatures increased, whereas oxygen functional groups showed an inversely proportional trend. We can classify the functional groups on the surface of charcoal fines with carbon chains, such as the C−C/C−H group, as hydrophobic; and those containing oxygen, such as C−O/C−O−C/C−OH, C−O, C=O and O−C=O, as hydrophilic [36]. The hydrophobic surface of charcoal fines improved with higher temperatures, attested by the increased contribution of C−C/C−H species dominating the material composition. 

Studies point out that greater hydrophobicity ensures greater polymer compatibility [34,37,38]. More hydrophobic materials result in better polymer filling and compatibility, evenly incorporating charcoal particles into polymer matrices and improving the tensile strength and flexion of biocomposites [39]. Thus, charcoal fines could reinforce polymer matrices due to their hydrophobicity, which can provide resistance to biocomposites.

### 3.1. Insights about the Use Charcoal in Polymerics Biocomposites

**Figure 7:**
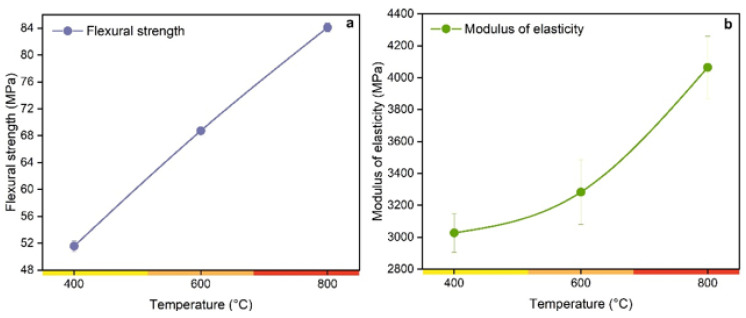
(**a**) Flexural strength and (**b**) modulus of elasticity of biocomposite produced with charcoal fines produced at different pyrolysis temperatures.

The effects of pyrolysis temperature on the properties of flexural strength and modulus of elasticity of biocomposites reinforced with charcoal fines are shown in Figure 7.

**Figure 8:**
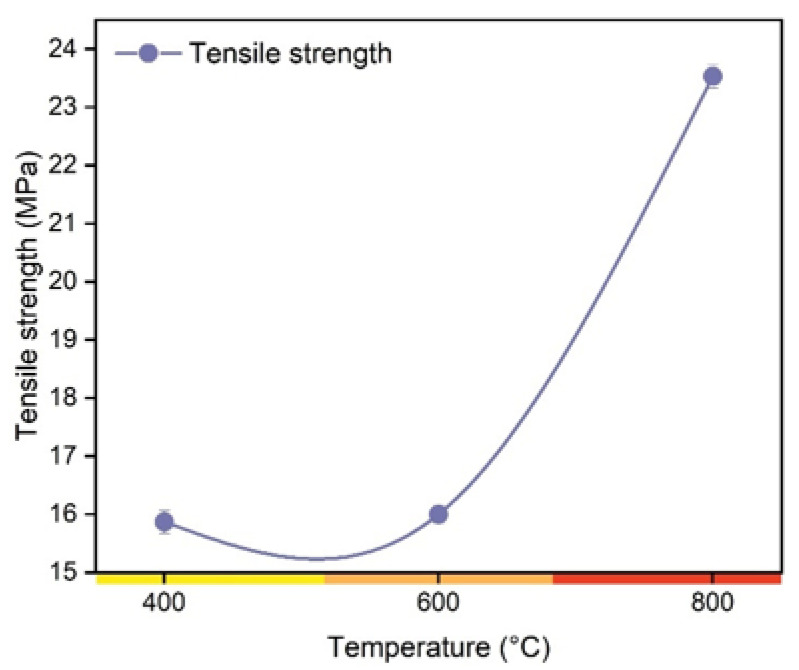
Tensile strength of biocomposites produced from charcoal fines obtained at different pyrolysis temperatures.

According to Figure 7, the pyrolysis temperature variation of the charcoal fines significantly affected the flexural properties and modulus of elasticity of the produced biocomposites. The resistance to reflection and the modulus of elasticity increase with increasing temperature (Figure 7). The flexural strength of the composites with charcoal fines °C was 51.6 MPa, a value lower than 84.1 MPa of the composites with charcoal at 800 °C. A higher flexural strength of a composite depends directly on the dispersion and filtration of the material particles in the resin used [6]. Higher pyrolysis temperatures result in charcoal fines with greater surface area, which favors greater resin fluidity in the charcoal pores, which causes physical/mechanical interlocking [5,6], which justifies the results of this study. Figure 8 illustrates the effect of pyrolysis temperature on the tensile strength of biocomposites produced with charcoal fines at different pyrolysis temperatures.

**Figure 9:**
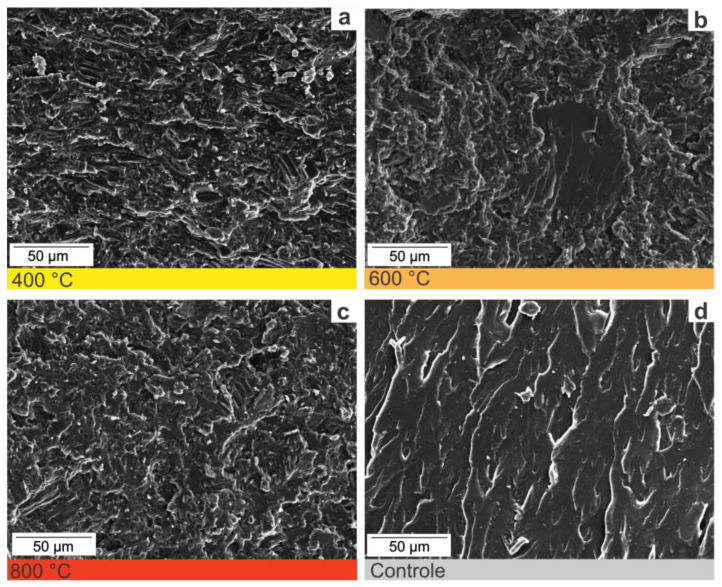
Micrographs obtained by scanning electron microscopy of biocomposites produced with charcoal fine samples synthesized at 400 (**a**), 600 (**b**), 800 °C (**c**), and control (**d**) in ×300 magnification.

It can be observed that the tensile strength values of the produced biocomposites increased to the increase in the pyrolysis temperature of the charcoal fines (Figure 8). The tensile strength of the fines produced at 400, 600, and 800 °C was 15.8 MPa, 16 MPa, and 23.5 MPa, respectively. As already reported in this work, the higher the pyrolysis temperature, the greater the hydrophobicity of the charcoal fines. This behavior may favor a better affinity between nonpolar polymer chains and charcoal fines, thus resulting in greater tensile strength [40]. Figure 9 shows the microstructure of the fractured surfaces of the biocomposites produced with charcoal and the composite made only with polyester resin.

Adding charcoal fines favored the formation of a rougher surface (Figure 9a–c) compared to the resin-only composite (Figure 9d). The biocomposites produced with charcoal fines showed reasonably good surface adhesion. There was no significant difference in the microstructure of the biocomposites produced at temperatures of 400 and 800 °C (Figure 9a,c). The polyester resin irregularly enveloped the charcoal fines. Generating a rough appearance on the surface of the biocomposite can be explained by the low interaction between fines and resin [41]. The roughness tends to decrease at a temperature of 800 °C, which may cause more significant interactions between the raw materials (Figure 9c) and consequently more excellent resistance of the biocomposite. These observations corroborate the results obtained in the mechanical tests (Figure 7 and Figure 8).

### 3.2. Practical and Policy Implications and Future Perspectives

Composites are technologically strategic materials with many applications in biomedicine, engineering, architecture, and dentistry, among others. However, nonbiodegradable petroleum-derived materials comprise several of these biocomponents. Researchers have widely investigated biocomposite development to offer a sustainable appeal to production systems since they are strategic, efficient, economically viable, and environmentally correct products which add value to charcoal residues. Currently, this waste lacks an adequate disposal or use due to its substantial amounts and the absence of government and social awareness. We believe no technical or scientific study have examined the practical potential of charcoal fines as reinforcement in polymeric matrices. Using this residue in polymeric biocomposites can reduce costs, dependence on fossil sources (plastics and other petroleum products) and, consequently, mitigate greenhouse gas emissions. 

Our results have practical and political implications, showing the potential benefits of reusing residual charcoal fines to produce biocomposites worldwide. The actions that can be developed based on our proposal favor the so-called industrial symbiosis, where waste from one sector becomes resources for the generation of products from another sector, contributing to resource efficiency and circular economy [42]. It is important to note that we present an innovative approach toward sustainability in this paper by including the concept of industrial symbiosis that is a part of the emerging field of industrial ecology, which demands resolute attention to the flow of materials and energy through local and regional economies. Industrial symbiosis engages traditionally separate industries in a collective approach to competitive advantage involving physical exchange of materials, energy, water, and/or byproducts [43]. The keys to industrial symbiosis are collaboration and the synergistic possibilities offered by geographic proximity. Moreover, industrial symbiosis [44] describes how a network of diverse organizations can foster eco-innovation and long-term culture change, create and share mutually profitable transactions, and improve business and technical processes. Thus, this perfectly complement the technical assessment of the biomass when processed under different conditions with the utilization of waste from one industry to be used by another industry to add value.

Our proposal contemplates two of the Sustainable Development Goals developed with the participation of world leaders and international non-governmental organizations. These are Goal 9, which aims to “Build resilient infrastructure, promote inclusive and sustainable industrialization and foster innovation”, and Goal 12, which aims to “Ensure sustainable production and consumption patterns”. The Brazilian National Solid Waste Policy (Law 12305/2010) provides for this action, encouraging waste reuse and valorization [45,46]. Brazil still needs to develop sectoral plans to reduce such waste accumulation, but national development planning or regulation still ignore most of these policies and instruments. Moreover, we should mention this study may impact society since biocomposites manufactured with charcoal fine-filling will benefit small and medium producers of this raw material. This study assists public management and companies in planning future goals toward charcoal waste, which will generate and develop new materials for various industrial sectors, such as civil construction, automotive, and biomedical, among others. 

## 4. Conclusions

Charcoal fines at 800 °C show good carbonic, porous, and hydrophobic results, making them a potential material for biocomposite production, which would allow a greater interaction and higher compatibilization with polymer matrices. The composites’ results show that the fines produced at 800 °C had better mechanical resistance. The search for innovations and use of polymeric biocomposites is increasing, and this study shows another possible use for these materials. We suggest future studies seeking optimizing the pyrolysis yields or their speed (flash pyrolysis) in order to obtain charcoal for polymeric matrices.

## Figures and Tables

**Table 1:** Tables should average percentage of functional states in C1s XPS spectra.

Sample	C=C, C−C, C−H (Aromatic, Aliphatic Carbon)	C−O, C−O−C, C−OH (Hydroxyl, Ether, Phenol)	C−O (Ketone)	C=O (Carbonyl)	O−C=O (Carboxylic Acid, Ester)
CV 400	62.1 ± 1.9	19.3 ± 0.9	10.8 ± 0.7	3.9 ± 0.3	3.95 ± 0.08
CV 600	72.1 ± 1.7	12.7 ± 2.5	7.5 ± 0.5	3.4 ± 0.2	4.3 ± 0.1
CV 800	70.4 ± 1.8	12.5 ± 1.5	7.7 ± 0.8	3.8 ± 0.2	5.7 ± 0.2

## Data Availability

The data presented in this study are available on request from the corresponding author.
